# *Citizen Sensors* for SHM: Towards a Crowdsourcing Platform

**DOI:** 10.3390/s150614591

**Published:** 2015-06-19

**Authors:** Ekin Ozer, Maria Q. Feng, Dongming Feng

**Affiliations:** Department of Civil Engineering and Engineering Mechanics, Columbia University, 500 W 120th St, New York, NY 10027, USA; E-Mails: mfeng@columbia.edu (M.Q.F.); df2465@columbia.edu (D.F.)

**Keywords:** Citizen Sensors, smartphone sensors, structural health monitoring, crowdsourcing, mobile networks, modal identification, ambient vibration

## Abstract

This paper presents an innovative structural health monitoring (SHM) platform in terms of how it integrates smartphone sensors, the web, and crowdsourcing. The ubiquity of smartphones has provided an opportunity to create low-cost sensor networks for SHM. Crowdsourcing has given rise to citizen initiatives becoming a vast source of inexpensive, valuable but heterogeneous data. Previously, the authors have investigated the reliability of smartphone accelerometers for vibration-based SHM. This paper takes a step further to integrate mobile sensing and web-based computing for a prospective crowdsourcing-based SHM platform. An iOS application was developed to enable citizens to measure structural vibration and upload the data to a server with smartphones. A web-based platform was developed to collect and process the data automatically and store the processed data, such as modal properties of the structure, for long-term SHM purposes. Finally, the integrated mobile and web-based platforms were tested to collect the low-amplitude ambient vibration data of a bridge structure. Possible sources of uncertainties related to citizens were investigated, including the phone location, coupling conditions, and sampling duration. The field test results showed that the vibration data acquired by smartphones operated by citizens without expertise are useful for identifying structural modal properties with high accuracy. This platform can be further developed into an automated, smart, sustainable, cost-free system for long-term monitoring of structural integrity of spatially distributed urban infrastructure. Citizen Sensors for SHM will be a novel participatory sensing platform in the way that it offers hybrid solutions to transitional crowdsourcing parameters.

## 1. Introduction

Structural health monitoring has attracted significant attention as the computational and technological environment matures. Vibration-based SHM has been explored for damage detection, model updating, performance assessment, and reliability estimation of civil engineering structures such as buildings and bridges (e.g., [1,2,3,4,5,6,7]), bringing new solutions to cope with aging and deteriorating urban infrastructure. Besides, the exponential growth of internet and smartphones has brought novel solutions to civil and earthquake engineering problems with citizen engagement [8,9,10,11]. Likewise, the widespread use of smartphones has produced a new potential source for vibration monitoring of civil infrastructure. State-of-the-art smartphone technology takes advantage of multiple embedded sensors to maximize the user experience and device productivity. Moreover, its advanced communication and networking capabilities enable the users to connect with each other or the web. A number of studies have discussed the possibility of using smartphones and citizen collaboration for SHM purposes [12,13,14].

Crowdsourcing has become popular over the last few years [15,16,17,18,19,20,21]. By definition, crowdsourcing is a collaborative problem-solving process with help from the community and volunteer participation, leading to a new understanding to Von Hippel’s user-oriented innovation concept [22]. In particular, with the rise of civic participation in a variety of platforms, innovative organizations have initiated new projects to make use of crowdsourcing as a low-cost or no-cost labor. Successful examples include commercial entrepreneurships such as Amazon’s Mechanical Turk, InnoCentive or nonprofit organizations such as Wikipedia. For instance, software engineering platforms use crowdsourcing as an access to technological progress [23,24,25,26,27,28,29]. Moreover, a wide range of research areas have benefitted from sourcing the crowd for environmental [30,31], geospatial [32,33,34], seismicity [35], and finally SHM studies [36]. In spite of the advantages offered by crowdsourcing, the data quality and accuracy need to be validated [37,38]. Likewise, machine learning methods might be utilized to detect false vibration measurements such as falling or defected phones and discard the flawed data accordingly [39].

These advancements have inspired the authors to develop a novel crowdsourcing-based Citizen Sensor System for SHM, which utilizes smartphone-embedded sensors for measuring structural vibration and defining sensor locations. In their previous study, the authors investigated the performance of smartphone accelerometers through a number of laboratory and field tests on civil engineering structures, and confirmed the usefulness of these sensors [40]. As a further step, this study aims at developing a novel crowdsourcing platform, which enables citizens to use their smartphones to measure structural vibration, transmit the data to an online server and process the data into a database automatically. The crowd incentives can be established through contests and rewards [41,42]. For example, the best identification results or participation above a certain sampling number could be rewarded to increase citizen encouragement. Another possibility is to utilize gamification strategies to convert the identification problem into an entertainment medium [43,44]. What is more, because the modal identification results may reveal post-event structural damage due to extreme events or aging, integrity and safety of urban infrastructure itself is a fundamental incentive that can mobilize people for crowdsourcing-based SHM. Pedestrians for bridges and occupants for office and residential buildings can be the target group for citizen sensors.

The proposed system includes a multilayered structure integrating mobile sensing and web platforms. An iOS (iPhone Operating System) smartphone application provides citizens with a tool for measuring structural vibration and submitting data wirelessly to a central server. The web-based server receives the citizen submissions, processes the vibration data and stores the processed data such as the identified modal properties (frequencies, damping ratios and mode shapes) as well as the vibration time history data. In this study, a crowdsourcing review is conducted to effectively formulate citizen experience and contribution. The platform developed in this study is then tested through field measurements on a bridge structure. A number of low-amplitude ambient vibration measurements with varied phone locations and coupling conditions are made to evaluate sources of uncertainties associated with citizen participation. Short-term individual data are collected to generate large-sized data and are averaged to compensate short measurement duration which is uncommon for SHM under ambient vibration. The results show that a smartphone-based system can produce valuable SHM information even with uncertainties associated with the citizen participation. What is more, integration with the web server enables modal identification in an online and automated manner.

This study lays a technical foundation for crowdsourcing-based, citizen-engaged SHM applications. Therefore, the case presented in this paper is a small-scale crowdsourcing problem discussing the issues related to citizen participation. The progress of Citizen Sensors for SHM will lead to a unique crowdsourcing example, because of its transitional descriptions due to the existing taxonomies. In other words, the presented platform will be the initial stage of a complex system which utilizes crowd participation, mobile sensing, and web services in a hybrid framework.

## 2. Multilayered Computer Platform

The goal of the computer platform developed in this study is to connect citizens with their smartphone sensors and a web-based server. The platform has a multilayered structure including the user, communication, and server layers, while each layer can be designed, implemented, and tested independently.

The user-side platform is based on the mobile devices used by the citizen participants. A smartphone application is developed to enable citizens to collect data with the smartphone-embedded sensors and transmit sensor data. Data transmission between the citizens and the server uses an existing cellular network or Wi-Fi and will not be discussed in this paper.

**Figure 1:**
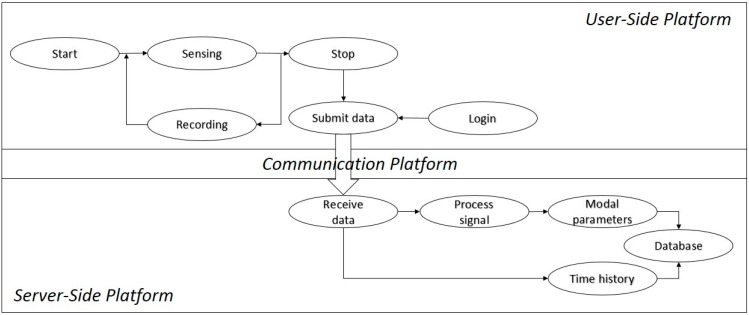
Integration scheme of system platforms.

The server-side platform receives, processes, and stores the measured vibration time-history data and processed results. The documents regarding system architecture scheme, requirements analysis can be found in [45]. Similarly, collaboration diagrams describing architectural design, class diagrams describing database design can be found in [46]. What is more, the software components and database tables are provided in [47]. Figure 1 shows the integrated, multilayered platform and its components. Details regarding user-side and server-side platforms are discussed within the following subsections.

### 2.1. User-Side Sensing and Acquisition

An iOS application named *Citizen Sensors for SHM* was developed as part of this study to enable citizens to measure vibration with their iPhone-embedded accelerometers and to submit the data to the server on Internet via a cellular network or WiFi connected to the Internet,. Xcode Version 6.1.1 is used for coding the application. Objective-C is used to develop the header and implementation files of the application. Cocoa Touch is the user interface framework which provides a wide set of classes for application development. The iOS application development is logically divided into three categories such as model-view-controller (MVC). With this approach, it is possible to build the computational background, design a user layout and connect these separate aspects with modular principles. In other words, MVC separates the application components in a modular way. In MVC approach, “Model” is involved in application data and methods, whereas “View” provides the user with interaction widgets. The third component, “Controller”, isolates the other two components from each other, controls the connection between them and updates both components based on received actions from “View” and data from “Model” [48].

**Figure 2:**
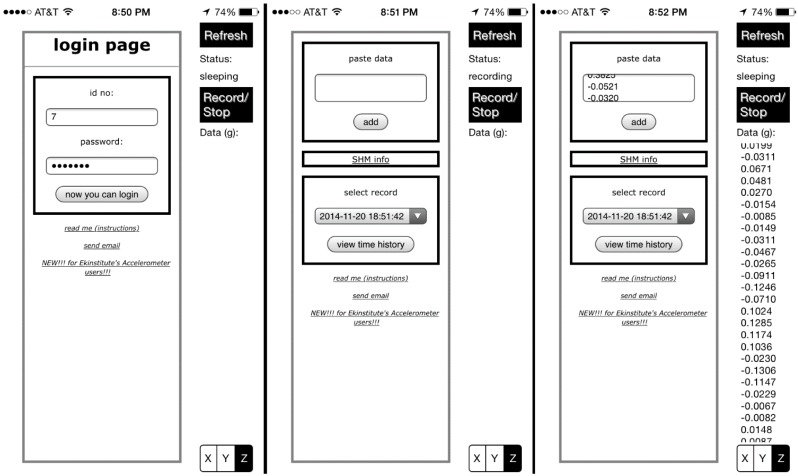
User login, recording, and submission screenshots, respectively.

In order to provide users with a simple interface, a single view application is chosen as the project template. The interface building element storyboard is utilized to set up interface objects, header (ViewController.h) and implementation (ViewController.h) file scripts are developed after interface objects and scripts are connected via the assistant editor. Basically, four interface objects are introduced to show the application status, activation button, acceleration time history column and the gateway to the server. Being generated by predefined object types such as UILabel, UIButton, UITextView, and UIWebView, these objects are introduced to the model via outlets and actions to display smartphone sensor data and receive user commands. Once the user touches the activation button object, the application requests acceleration data from the phone’s accelerometers at a sampling rate (such as 100 Hz, which is sampling frequency’s upper limit for old generation iOS devices [49]). This means that the application is capable of identifying modal frequencies up to Nyquist frequency, 50 Hz, which is equal to the half of sampling frequency. The acquired data are accumulated in a temporary variable and transferred to the acceleration time history column once the button is repressed. The user simply logs in and uploads the acceleration time history data to the server via the web view object. Figure 2 shows three screenshots of the iOS application interface, which enables the users to interact with the smartphone sensors and the server. The application *Citizen Sensors for SHM* is currently available at the iTunes Store [50]. Further sources regarding the iOS application development can be found in [51,52]. This application is developed for iOS, and can be extended to different mobile operating systems such as Android, Windows Mobile, and Blackberry 10 in the future, provided that smartphone models have embedded accelerometers.

### 2.2. Server-Side Processing and Database

The web-based server-side platform receives the acceleration time history data measured by the citizens at a structure, processes the data to identify the modal properties of the structure (such as the modal frequencies) which are correlated with the structural health conditions as shown in previous studies, and stores the results as well as the raw data. An administrator may be granted with an online access to the data in the server. A number of computer languages are used to build the platform. PHP (formerly Personal Home Page, recently PHP: Hypertext Preprocessor) is used as the main scripting language throughout webpage development process. The database is constructed with MySQL (SQL: Structured Query Language), and automatically updated by MySQL codes embedded in PHP scripts. In order to produce a web interface with a user friendly design, HTML (HyperText Markup Language) and CSS (Cascading Style Sheets) scripts are developed. A web platform was built on a server hosted by a commercial web-hosting service and is accessible online [53].

**Figure 3:**
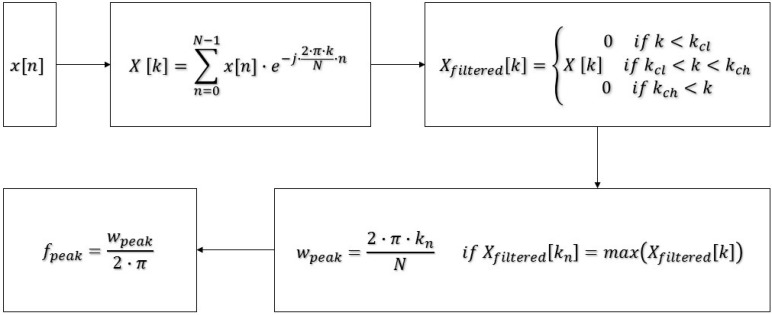
Digital signal processing operations applied on the server-side.

The system is designed to provide an online SHM environment which is capable of being used by multiple users and multiple structures at the same time. The system receives the acceleration time history from the users, conducts discrete Fourier transform (DFT) analysis, determines peak frequency and stores the input and the output data with the submission details such as measurement date, record number, and user identification number. Figure 3 summarizes the digital signal processing applications implemented in server-side to apply band-pass filter to the acquired raw data and compute the natural frequency based on the DFT results [54].

The web users are divided into two categories, citizens and administrators. Citizen accounts are created, managed and given access to records by the administrator and do not have permission to modify database except vibration data submission. Other than that, citizens have access to view the information related to their account. A new account is created with an automatically generated identification number. Similarly, once the platform is ready to be used at massive scales, citizens will be provided with randomly generated passwords. In other words, the platform will not store any personal information until privacy, anonymity, and security issues are comprehensively dealt with. Furthermore, volunteers will be offered to opt-out to avoid violation of privacy. Administrators can activate or deactivate citizen accounts, have access to the data provided by citizens such as analysis results or vibration time history records either for a specific structure or multiple structures. This ability provides the potential to develop a further relationship between long-term monitoring records and reveal correlations between common environment-induced parameters (e.g., wind, temperature changes, earthquakes) to generate big data. However, because the proposed platform has recently been initiated, big data analytics is a long term goal and is not addressed in this paper.

**Figure 4:**
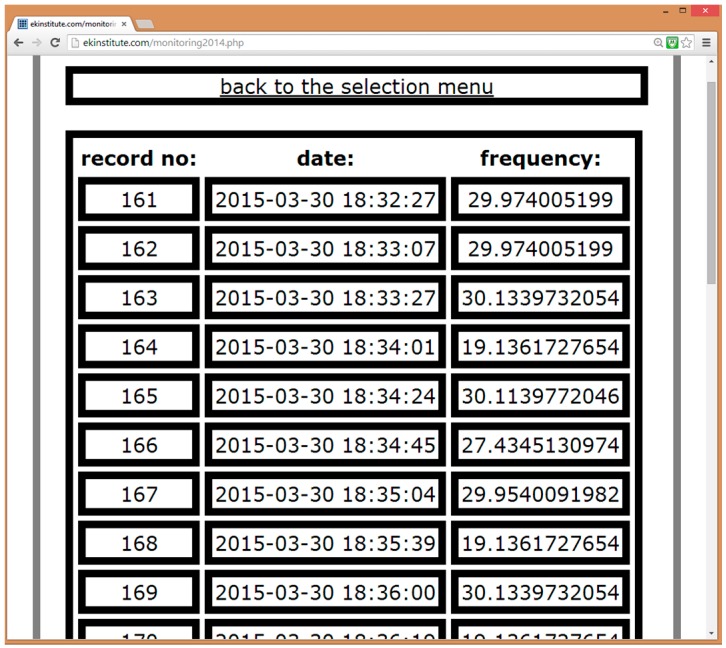
Screenshot from the web interface showing the SHM results page.

The web platform is built on PHP scripts referencing each other according to the submission or monitoring process. The hierarchical script reference order for a user starts with the index as the first step, login as the second step, view of user’s own monitoring results and previously submitted data, or addition of new data as the third and the last step. Viewing one’s own monitoring results on landmark structures (e.g., Eiffel Tower, Golden Gate Bridge) can be one of the incentives that motivate citizens to participate as crowdsourcers. The administrator account has the same hierarchy except its access is extended to the entire database, and can delete or assign new user accounts. Further details regarding PHP-MySQL integrated web development are referred to [55]. Figure 4 is an example of the interface showing the overall measurement results at a specific structure, including the record number, measurement date and the structure’s natural frequency in Hz identified from the vibration measured at the structure.

## 3. Crowdsourcing

Another important component of *Citizen Sensors for SHM* is citizens, therefore utilizing citizens’ enthusiasm for problem solving is crucial. In other words, crowdsourcing, basically collaborating with citizens, has an important role in system performance as well as the computer platform. For the purpose of understanding current crowdsourcing methodologies, a number of different approaches are evaluated.

Howe was the first to diagnose upcoming low-cost labor and production model for the industry, society and more [15]. Crowdsourcing, as a problem solving paradigm, was one of the actors replacing conventional, static, individual approaches with a novel, online, distributed model [16]. Like, the Internet, open source, and others, crowdsourcing—a virtual community—was one of the collaboration models identified by Albors *et al.* [17], and it was able to “create value for the general public [18]” even without a profit incentive.

Stemming from multiple theoretical foundations such as value chain, auction, motivation crowding, organizational learning, cognitive evaluation, transaction cost, strategic management, innovation and game theory [19], a mutual agreement regarding crowdsourcing definition still has not been established. Albors *et al* [17] presented a taxonomy which classifies collaboration alternatives according to social and information connectivity. Schenk and Guittard [20] distinguished crowdsourcing from related concepts such as open innovation, user innovation and open source software, classified different crowdsourcing practices, and discussed a number of opportunities and threats. Eventually, Estelles-Arolas and Gonzalez-Ladron-de-Guevara collected 32 different definitions, investigated their commonly-agreed aspects, and came up with a comprehensive definition based on the trend of existing studies [21]:

“Crowdsourcing is a type of participative online activity in which an individual, an institution, or company proposes to a group of individuals of varying knowledge, homogeneity and number, via a flexible open call, the voluntary undertaking of a task. The undertaking of the task, of variable complexity and modularity, and in which the crowd should participate bringing their work, money, knowledge and/or experience, always entails mutual benefit. The user will receive the satisfaction of a given type of need, be it economic, social recognition, self-esteem, or the development of individual skills, while the crowdsourcer will obtain and utilize to their advantage what the user has brought to the venture, whose form will depend on the type of activity undertaken.”

Based on the preexisting crowdsourcing definitions and classifications, the authors attempted to develop an SHM-oriented crowdsourcing model to receive smartphone sensor data via citizen contribution. A crowdsourcing model can be prepared by setting the proper actors and their corresponding actions. A robust classification defines crowdsourcing actors as “the crowd”, “the initiator”, and “the process” [21]. “The crowd” element is defined by (1) “who forms it”, (2) “what it has to do”, and (3) “what it gets in return” [21]. Similarly “the initiator” description must reveal (1) “who it is”, and (2) “what it gets in return” [21]. Finally, “the process” refers to “the type of process, the type of call, and the medium used” [21]. Likewise, crowdsourcing actors can be distributed into three groups: individuals as crowd participants, beneficiary company/institute, and the platform connecting individuals and beneficiaries [20]. Crowdsourcing tasks can be divided into three groups: “cognitive dimension”, “nature of incentives” and “benefits of crowdsourcing” [20]. What is more, based on the individual value’s importance with respect to the community, crowdsourcing can be either “integration-based” or “selection-based” [20]. Similarly, crowdsourcing dimensions can be described by agents such as “provider”, “mode”, “ownership”, and “motivation and incentive” [19]. In addition, crowdsourcing can be divided into elements such as “components”, “processes”, and “actions” [19]. Finally, the future crowdsourcing problem will evolve due to different perspectives such as “participant”, “organization”, and “system” [19].

Based on these foundations, the authors formulated the proposed crowdsourcing model with three actors [21], groups [20], or perspectives [19]: citizens, administrators, and web platform. Citizens herein are described as people who are motivated to take measurements of structures (such as buildings and bridges) and submit the data with their smartphones. Likewise, administrators’ motivation is to collect the best available vibration data and maximize structural system identification efficiency and accuracy. Finally, the process will involve mobile sensing, submission, server acquisition, digital signal processing, and database storage. The proposed system can be constructed on a combination of “integration-based” and “selection-based” crowdsourcing, since every participant is likely to have a different contribution accuracy, yet compose a strong, integrated platform when combined together [19,20,21].

In order to apply these crowdsourcing concepts to citizen-engaged smartphone-based structural health monitoring, a number of uncertainties causing variation in measurement results must be studied. Basically, these can be divided into (1) user-related; (2) hardware-related; and (3) structure-related uncertainties. User-related uncertainties can stem from a variety of different issues including users’ understanding of the crowdsourcing problem and platform, third-party accessories attached to their smartphones, and the time and quality of their measurement. Hardware-related uncertainties are mainly due to the model/performance of the sensors and CPU’s embedded in the users’ smartphones. Structure-related uncertainties can be caused by different vibration loading patterns including ambient vibration, operational vibration and extreme events (such as earthquakes). Considering these uncertainties, the authors specified crowdsourcing parameters including the vibration loading type, the smartphone model, the phone-structure coupling, the phone position, and measurement duration.

Finally, to provide the connection between citizen sensors and crowdsourcing, it is essential to understand the potential of smartphone sensors, with an emphasis on participatory sensing and mobile crowdsourcing aspects. For this purpose, a taxonomy discussing mobile crowdsourcing applications is taken as a reference, which defines a crowdsourcing solution in terms of its web-extension, involvement, data wisdom, contribution quality, incentives, human skill, sensors, and location [56]. The mobile crowdsourcing taxonomy characteristics are discussed from a citizen-engaged SHM perspective below:

### 3.1. Sensors

Smartphone sensors may not only serve crowdsourcing-based SHM with sole vibration data, but also enable citizens to obtain “smarter” measurements. For example, orientation errors due to smartphone placement can be corrected instantaneously by utilizing smartphone gyroscope. GPS and magnetometer data provides the server with the measurement location and direction that can be used to match the phone position with the structure and avoid submissions from false locations. Server-side workload can be reduced, and signal processing speed can be extensively increased by using smartphone processors as components of a distributed computing platform. If applicable, structural nodes can be assigned information features (e.g., barcodes, matrix codes) and can be automatically detected by smartphone cameras. Nearby excitation sources such as vehicles can be detected with the microphones, and their effects can be classified accordingly. If the environment is rich in participants, the devices can be synchronized with Bluetooth or Wi-Fi connection, and simultaneous data can be gathered from multiple channels.

### 3.2. Data Wisdom, Contribution Quality, and Web-Extension

Combining the mobile features [50] with the web server [53], it is possible to improve the crowdsourcing value by converting individual submissions into collective data as a means of data wisdom. In particular, averaging collective Fourier spectra will improve the individual results by discarding the noise. Thanks to a central platform with a structured database, heterogeneous and homogeneous vibration data can be organized, mined and structural features can be extracted even if the datasets involve high complexity.

### 3.3. Human Skill and Incentives

The way crowdsourcing-based SHM receives contributions is a mixture of labor and visual human skills which is ideally reduced as platform improvement progresses. These skills (adjusting device’s position, coupling conditions, sampling duration *etc.*) can be improved with motivation sources or educational tools (e.g., demos, instructions, user manuals). There is a wide variety of incentives that can be utilized such as receiving awards or safety (monetary, service), gamification (entertainment), and social responsibility (ethical). For example, the identification problem can be gamified such that the most accurate citizen submission can be rewarded. Likewise, a threshold can be set, and the citizens who have significant contribution can be acknowledged and honored. The platform can be linked to social media for advertisement and can attract those attention who are likely to participate in a novel crowdsourcing platform.

### 3.4. Involvement

By nature, crowdsourcing-based SHM involves many challenges due to its complicated structure. The quality of the vibration data depends on citizen’s intuition as well as the sensor quality. With the help of proper participatory sensing and mobile crowdsourcing strategies, though, citizen-induced error can be minimized. At this stage, Citizen Sensors for SHM resembles a hybrid crowdsourcing platform with participatory and opportunistic components. In other words, the participatory aspect is characterized by the smartphone user’s skills, whereas the opportunistic aspect basically relies on computer and sensor properties. Using all of the hardware and software capabilities to the best extent, the mobile crowdsourcing problem can partially be reduced from participatory to opportunistic, which differs from classical crowdsourcing approaches by taking advantage of mobile crowdsourcing tools [56].

To summarize, the crowdsourcing-based SHM presented in this study is already capable of using accelerometers, can be provided with further sensor and location services, and has the web extension, which are some of the mobile crowdsourcing fundamentals. As the platform is improved with the new sensors, computational tools, and services, the majority of the human skills will be replaced with sensor data. Moreover, many different incentives can be created depending on society’s and urban infrastructures’ needs. In addition, the platform presents a unique crowdsourcing solution in the way it combines participatory and opportunistic involvement, individual and collective data wisdom, heterogeneous and homogeneous contributions in a transitive manner. Table 1 presents the prospective Citizen Sensors for SHM (CS4SHM)’s characteristics with the taxonomy and the examples provided by [56].

**Table 1:** Mobile crowdsourcing taxonomy, examples, and crowdsourcing-based SHM [56].

Application	Web	Involvement	Data	Contribution	Incentives	Skill	Sensors	Location
Gigwalk.com	Yes	Participatory	Individual	Heterogeneous	Monetary	Labor	Camera	Yes
CityExplorer	No	Participatory	Collective	Homogeneous	Entertainment	Visual	Camera	Yes
PotHole	No	Opportunistic	Collective	Homogeneous	Ethical	Non	Vibration	Yes
CS4SHM	Yes	Both	Both	Both	Multiple	Multiple	Multiple	Yes

## 4. Case Study: Monitoring of a Pedestrian Link Bridge

Field measurements are conducted in order to evaluate the capability of the *Citizen Sensor* system developed in this study. The purpose of these tests is to evaluate the integrated SHM system with firsthand experience. Moreover, the system is tested to see if it can produce valuable modal identification results for SHM purposes. In other words, accuracy of modal identification is important for SHM, since they are highly correlated with structural integrity. Finally, the citizen-induced uncertainties such as coupling, positioning, and duration are studied. Therefore, implementation of the proposed platforms is tested on a pedestrian bridge structure which is widely accessible by citizens.

**Figure 5:**
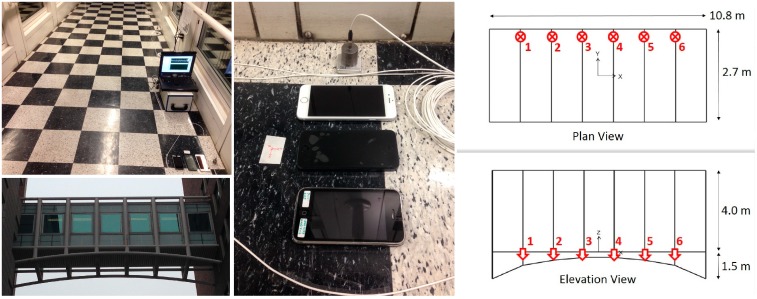
Inner and outer views, dimensions, and sensor layout of the pedestrian link bridge.

The structure is an 11-m single span steel arch bridge, which serves as a passage between two multistory buildings. Because bridge flexibility is expected to be higher than adjacent reinforced concrete multistory buildings, dynamic effects due to these adjacent structures are not considered. Figure 5 shows the inner and outer views of the bridge structure, dimensions, and sensor layout for reference measurements.

The bridge is instrumented with six high-quality reference piezoelectric accelerometers of model 393B04 PCB Piezotronics. The reference accelerometers are fixed via double-sided adhesive tape. The data is transmitted through cable connection, and acquired by a data acquisition system (National Instruments SCXI-1531) synchronously at a sampling rate of 100 Hz. The measurements are sequentially conducted at nighttime, to minimize passenger-induced vibrations and obstructions in the test procedure. In other words, the ambient vibration is dominated by low-amplitude environmental effects such as wind, rather than walking-induced structural input. Then, the Frequency Domain Decomposition (FDD) method is used to conduct modal identification and obtain modal frequencies and mode shapes as the reference. Afterwards, a number of tests with changing coupling conditions and sensor locations are conducted to compare smartphone measurements with reference measurements and evaluate smartphone sensor behavior under different citizen-induced conditions.

### 4.1. Measurement, Data Processing and Modal Identification

In order to determine modal characteristics of the bridge structure, high-quality, synchronous, multichannel vibration data is acquired and processed as the reference. The accelerometers are oriented in the vertical direction, and are equally spaced spanning the longitudinal direction, as shown in Figure 5. Therefore, acceleration time histories at six different locations are obtained under ambient vibration and processed with FDD method to determine modal frequencies and mode shapes in vertical direction. By discretizing multi-channel vibration data in the frequency domain, arrays of spectral values are generated for each discrete frequency step. Singular value decomposition of these matrices will result in eigenvalues and eigenvectors, which corresponds to the singular values and modal displacements as a function of frequency. These functions are used to determine the modal frequencies and mode shapes. For brevity, the first three modes in vertical direction are considered, whereas lateral, longitudinal and torsional, and higher modes are not discussed. Further details regarding FDD method can be found in [57].

**Figure 6:**
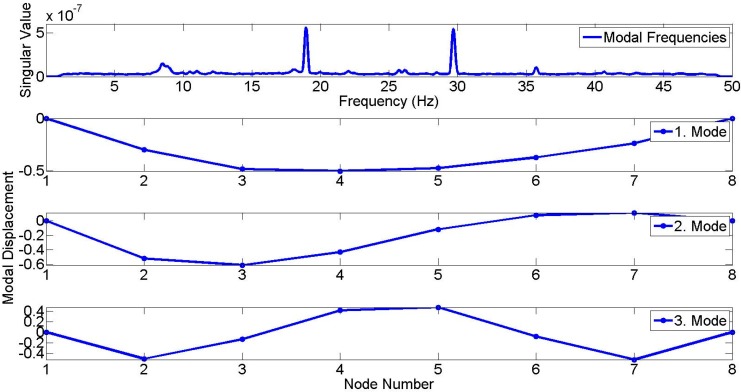
1st, 2nd, and 3rd modal frequencies (8.46, 18.95, 29.67 Hz) and mode shapes from FDD by reference accelerometers.

Figure 6 shows the singular value spectra obtained from FDD analysis. It is observed that the second and the third modes dominate the vibration characteristics, and spectral value due to first mode is relatively small. Accordingly, the first, second and third modal frequencies are identified as 8.46, 18.95, and 29.67 Hz, respectively. The mode shapes corresponding to the first, second and third modes are presented in Figure 6. According to these mode shapes, modal displacements due to the first, second, and third mode are maximized at Node 4, Node 3, and Node 2, respectively.

### 4.2. Uncertainties Associated with Citizen Participation

After the system’s dynamic characteristics are determined with the advanced reference system identification tools, smartphone measurements are taken to compare their performance with the reference results. Based on the crowdsourcing discussions presented in Section 3, a number of tests are conducted to characterize smartphone performance under changing user-related conditions. These conditions include different coupling conditions, as well as location effects. These parameters are evaluated for different smartphone generations such as Smartphone 1, Smartphone 2, and Smartphone 3 which corresponds to iPhone 3GS, iPhone 5, and iPhone 6, respectively. In keeping with the crowdsourcing discussions in Section 3, a number of principles are developed to maximize citizen engagement. These principles characterize the uncertainties and challenges of a potential crowdsourcing-based SHM methodology such as:

(1) Smartphone location and orientation might change according to smartphone users’ initiative.
(2) Smartphone coupling conditions might vary according to external accessories or surrounding material.
(3) Measurement duration can extensively vary according to the users’ motivation.
(4) Users should not be subjected to additional charges for data submission and therefore are allowed to prefer different communication platforms to submit data (wireless, cellular, *etc.*).

Considering these principles, a number of regulations are made to decrease the level of uncertainty. For instance, for this structure, unless external mounting instrumentation is used, the only convenient device orientation is the z-direction, with the phone’s rear side facing the bridge deck surface. Therefore, other device orientation effects are not considered as influential parameters. For modes other than the vertical ones, the mobile application allows users to adjust the sensing direction. Moreover, to allow user benefit from smartphone communication capabilities in the preferred way, one can either submit data right after acquisition or keep the time history as a text file until preferred communication tools are available. What is more, citizens are not expected to spend a long time on the bridge; instead, they stop by for a limited amount of time, not more than a few minutes. Therefore, data submissions are received in small data packages and every one-minute data is presented as a sample. The strategy to take advantage of crowdsourcing presented herein is to keep citizen comfort high and receive large numbers of samples from a large-sized community, rather than being dominated by few users. Eventually, the monitoring results will rely on the society as a whole rather than a small number of individuals.

In order to implement these principles into the developed platform, a number of different tests are conducted to evaluate these crowdsourcing effects on sensing performance. Table 2 presents the parameters of six different tests which vary in measurement location and coupling conditions. Test 1, Test 2, Test 3, and Test 4 compare smartphone sensor performance under different coupling conditions, whereas Test 3, Test 5, and Test 6 observe the difference between different sensor locations. Therefore, in Test 1, Test 2, Test 3, and Test 4, smartphones are either attached to the bridge floor with double-sided adhesive tapes, or set free to move with or without a smartphone case, or contained in a bag. Test 3, Test 5, and Test 6 keep the coupling conditions constant while sensor location is different such as midspan, one-third span, and one-sixth span.

**Table 2:** Field measurement with different sensor locations and coupling conditions.

Test No	Time (min)	Sensor Location	Coupling Conditions
1	40	Mid-span	Adhesive Taped
2	40	Mid-span	Free to Move–With Case
3	40	Mid-span	Free to Move–No Case
4	40	Mid-span	Free to Move–In Bag
5	40	One-third Span	Free to Move–No Case
6	40	One-sixth Span	Free to Move–No Case

As mentioned before, to maintain citizen patience and motivation throughout measurements, duration of a sample is set equal to one minute. This is contradictory with the conventional ambient vibration measurement practice, because long-duration measurements are more reliable for removing random noise. While the measurement duration of each citizen is relatively short (*i.e.*, one minute), a significant number of submissions from many citizens are expected to achieve reliable measurement results.

**Figure 7:**
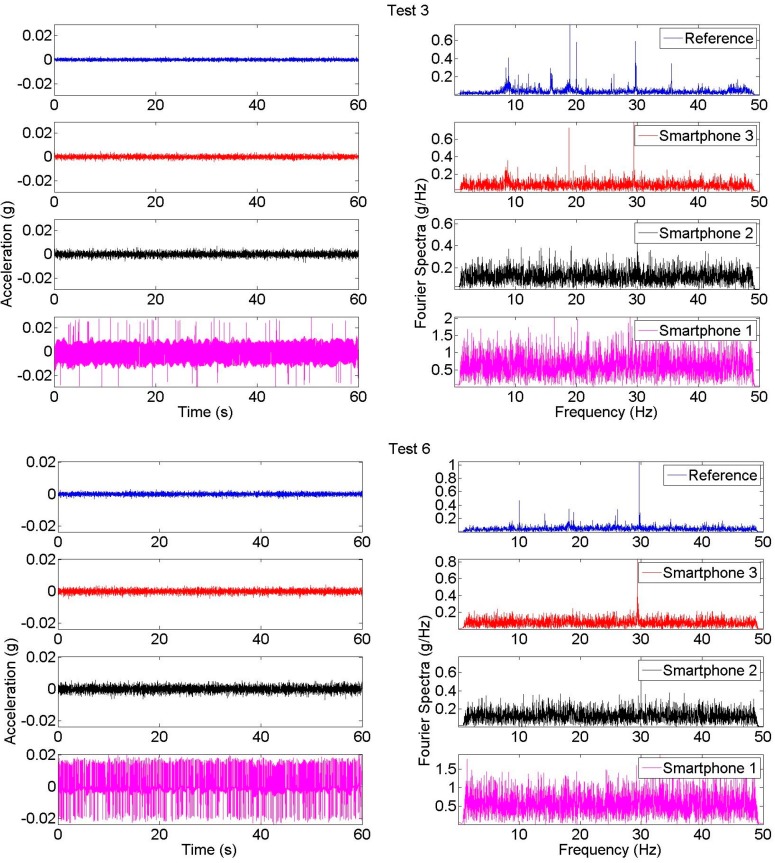
Acceleration time histories and Fourier spectra samples from Test 3 and Test 6.

In this study, it is observed that most of the smartphones measured the bridge’s ambient vibration reasonably well. Figure 7 shows the time history and Fourier spectra of two samples obtained during Test 3 and Test 6. According to Figure 7, similar to the reference modal identification results, dominant peaks are located at 20 and 30 Hz whereas the first modal peak is less significant around 8.5 Hz. According to the time histories and Fourier spectra, it is seen that vibration signal amplitudes change according to the smartphone generation. For instance, it is seen that the reference sensor has the lowest amplitude, whereas amplitude increases as the smartphone model gets older. Likewise, it can be observed that Smartphone 1 measurements have very high amplitudes in the time domain, and high spectral values in the frequency domain. These coincide with the relatively low sensor quality of Smartphone 1 discussed in [40] and the measurements are corrupted due to high level of noise. In other words, there is a correlation between the measured amplitudes and the sensitivity levels of accelerometers which are 18, 1, and 0.24 mg/digit for Smartphone 1, Smartphone 2, and Smartphone 3, respectively. Detailed information for Smartphone 1, Smartphone 2, and Smartphone 3 sensors can be found from the datasheets of the accelerometers LIS331DL (ST Microelectronics), LIS331DLH (ST Microelectronics), and BMA280 (Bosch Sensortec), respectively.

Another way to observe noise effects is that the Fourier spectra of Smartphone 1 are extremely broad-band, which resembles a typical white noise spectra and does not reflect structural vibration characteristics. Looking at the newer smartphone generations, Smartphone 2 and Smartphone 3, the smartphone signal amplitude is greatly reduced as the noise level reduces and the structural peaks become more significant as the smartphone generation gets younger. A similar pattern can be observed by evaluating the Arias intensity of acceleration signals which is a measure of signal energy [11,58] and is correlated with the area under the vibration signal.

**Figure 8:**
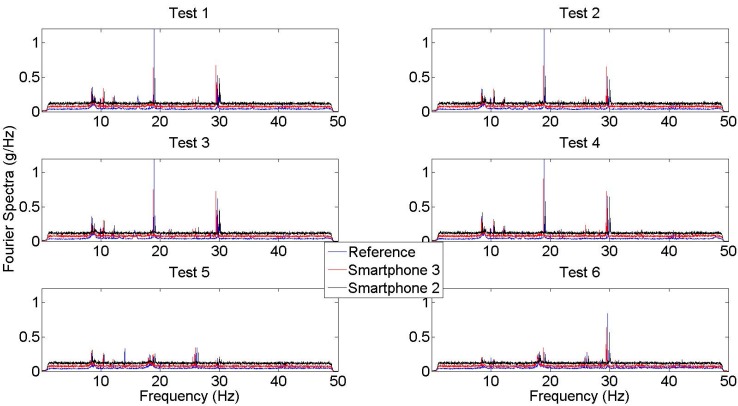
Fourier spectra from the average of 40 samples for Test 1–6.

A long measurement duration (several minutes) is desired to measure low-amplitude ambient vibration of a real structure, in order to average out random noise. When engaging a large number of citizens to do the measurement, however, even a short measurement duration from each citizen might be sufficient, as long as the total duration of measurement is sufficiently long. This study tested this by collecting and averaging 40 individual samples as in Figure 7. Averaged Fourier spectra curves are obtained with no overlapping between samples. Each one-minute sample is transformed into the frequency domain with a frequency resolution equal to 0.0167 Hz. An overall dataset corresponds to 40 samples and a total duration of 40 min, because the samples are processed with no overlapping. Figure 8 shows the averaged spectral curves obtained from different tests. Compared with the spectra obtained from a single sample, it is observed that noise level is significantly reduced, and peaks representing modal frequencies are much more significant.

Comparing averaged spectra of different tests, it is observed that Test 1, Test 2, Test 3, and Test 4 spectra have the same characteristics, whereas there is a significant reduction in 1st and 2nd modal frequency peaks in Test 5 and Test 6. This reveals that the proposed coupling conditions did not have a significant effect on spectral values since Test 1–4 has the same location at mid-span. The difference in Test 5 and Test 6 is due to the location difference between tests. For instance, Test 3 and Test 4 location corresponds to the location of one-third and one-sixth span unlike other tests.

**Figure 9:**
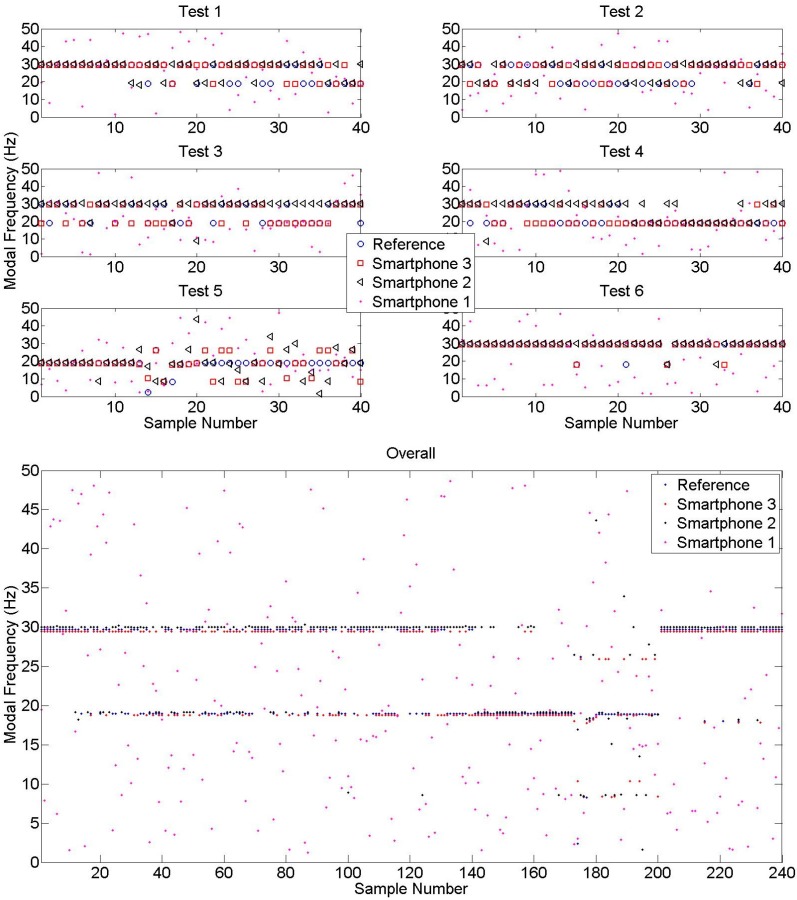
Identified frequencies obtained from different samples.

To summarize overall modal identification performance, the peak frequency values obtained from a large number of samples are plotted in Figure 9. It can be observed that Smartphone 2 and Smartphone 3 modal identification results match reference measurements with a significant accuracy, whereas Smartphone 1 results do not provide any modal information as they are masked by the high noise level. Moreover, the results of Test 1, Test 2, Test 3, and Test 4 show that Smartphone modal identification results are accurate even under challenging coupling conditions (*i.e.*, free to move, with case, in bag). Likewise, modal identification results obtained from different locations still reflect structural characteristics, but the quality may change according to the modal displacement of the measurement location. For instance, peaks obtained from Test 5 identify the second mode to a better extent, whereas third mode is more significant on Test 6 results. The reason is that second and third modal displacement is maximized at testing locations, which are Node 5 and Node 6, respectively. Finally, collecting all samples together, looking at Figure 9, the second and third modal frequencies are identified occasionally, whereas the first mode is identified in a small number of samples.

**Figure 10:**
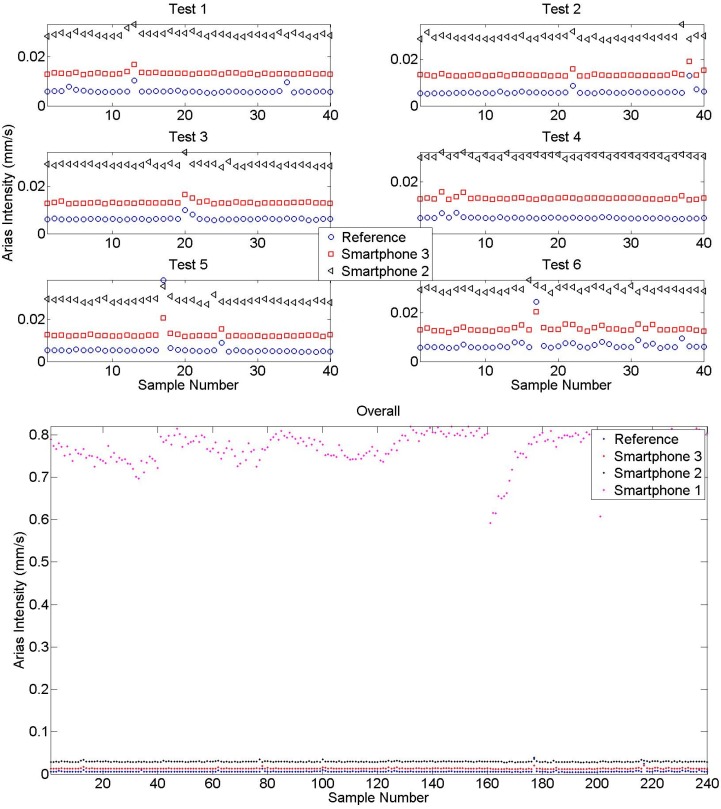
Arias intensities obtained from different samples.

In Figure 10, Arias intensity values are plotted to observe the energy difference between reference and smartphone sensors. The overall figure shows that there is a great difference between intensity levels obtained by Smartphone 1 and others. As explained before, the high level of noise results in overestimation of energy in measurements, especially under low intensity ambient vibrations [11,40]. Therefore, Smartphone 1 intensity is not demonstrated in Test 1–6 results to increase the graph resolution. According to the intensity values obtained from different tests, it is observed that the new generation Smartphone 3 performs better than Smartphone 2 due to the increased sensor sensitivity level. However, both results are considerably accurate as a means of modal identification under ambient vibration.

Table 3 summarizes the performance of smartphone sensors in terms of identified modal frequencies. The performance difference between different tests was insignificant and therefore not presented as individual results. The modal frequencies obtained from each averaged spectra are compared with FDD results, and error values are presented. The results show that Smartphone 1 is incapable of identifying modal frequencies. In contrast, new generation smartphones achieve highly accurate results such as 1.30%, 1.06%, and 1.05% for Smartphone 2; 0.71%, 0.79%, and 0.81% for Smartphone 3.

**Table 3:** Identified modal frequencies.

Sensor	*f*_1averaged_	Error (%)	*f*_2averaged_	Error (%)	*f*_3averaged_	Error (%)
Reference	8.48	0.24	18.97	0.11	29.68	0.04
Smartphone 3	8.40	0.71	18.80	0.79	29.43	0.81
Smartphone 2	8.57	1.30	19.15	1.06	29.98	1.05
Smartphone 1	-	-	-	-	-	-

Table 4 shows mean and standard deviation values obtained from each test with 40-sample sets in terms of peak vertical acceleration (PVA) and Arias intensity. According to these results, it is seen that PVA and Arias intensity increases for older generation smartphones. For instance, the PVA value for reference sensors is close to 0.0038 g whereas Smartphone 1, Smartphone 2, and Smartphone 3 values range around 0.0045, 0.0066, and 0.0294 g, respectively. Similarly, Arias intensity values are approximately 0.006, 0.013, 0.029, and 0.75 mm/s for reference, Smartphone 1, Smartphone 2, and Smartphone 3, respectively. As indicated previously, such difference is likely to stem from the sensitivity level of each sensor. For example, compared with other sensors, low quality Smartphone 1 accelerometer’s amplitudes are extremely higher in terms of PVA and Arias intensity. The PVA and Arias intensity difference between different generations show that smartphone performance, in terms of amplitude, varies significantly according to the smartphone model unlike frequency domain performance. What is more, it is observed that the intensity level is not subjected to change throughout different samples, which means, smartphone sensors’ performance is stable over time.

**Table 4:** Peak vertical acceleration (PVA), Arias intensity mean and standard deviation values.

Test No	Sensor	*PVA*_μ_ (g)	*PVA*_σ_ (g)	*AI*_μ_ (mm/s)	*AI*_σ_ (mm/s)
1	Reference	0.0041	0.0030	0.0061	0.0010
	Smartphone 3	0.0044	0.0004	0.0133	0.0007
	Smartphone 2	0.0064	0.0005	0.0293	0.0010
	Smartphone 1	0.0278	0.0017	0.7455	0.0202
2	Reference	0.0036	0.0032	0.0060	0.0013
	Smartphone 3	0.0046	0.0010	0.0134	0.0011
	Smartphone 2	0.0067	0.0006	0.0297	0.0012
	Smartphone 1	0.0286	0.0022	0.7718	0.0218
3	Reference	0.0031	0.0008	0.0063	0.0007
	Smartphone 3	0.0044	0.0007	0.0131	0.0007
	Smartphone 2	0.0065	0.0006	0.0292	0.0010
	Smartphone 1	0.0289	0.0022	0.7710	0.0206
4	Reference	0.0031	0.0009	0.0061	0.0005
	Smartphone 3	0.0045	0.0006	0.0136	0.0006
	Smartphone 2	0.0068	0.0007	0.0299	0.0006
	Smartphone 1	0.0297	0.0020	0.7952	0.0210
5	Reference	0.0042	0.0048	0.0061	0.0053
	Smartphone 3	0.0044	0.0013	0.0125	0.0014
	Smartphone 2	0.0067	0.0009	0.0290	0.0014
	Smartphone 1	0.0308	0.0032	0.7560	0.0622
6	Reference	0.0049	0.0046	0.0069	0.0030
	Smartphone 3	0.0047	0.0007	0.0136	0.0014
	Smartphone 2	0.0065	0.0005	0.0296	0.0010
	Smartphone 1	0.0307	0.0023	0.7737	0.0487

**Figure 11:**
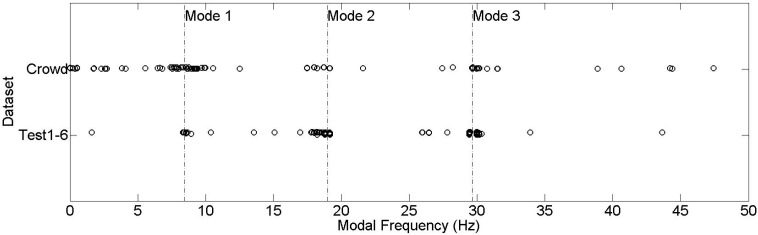
Modal identification results from Test 1–6 and crowdsourcing.

Finally, after investigating the citizen-induced parameters, student volunteers are assigned to test the crowdsourcing-based SHM platform. 135 samples are received, automatically processed, and the identification results are inserted into the web database. Figure 11 shows the distribution of crowdsourcing-based submission results compared with the results obtained from Tests 1–6. The distribution results show that there is a higher dispersion in crowdsourced identified frequencies, yet there is a similar trend with the Test 1–6 results which are conducted in a relatively controlled environment. Moreover, it can be observed that the crowdsourcing-based results tend to identify the 1st mode more often, whereas Test 1–6 results are concentrated on the 2nd and the 3rd modes.

**Figure 12:**
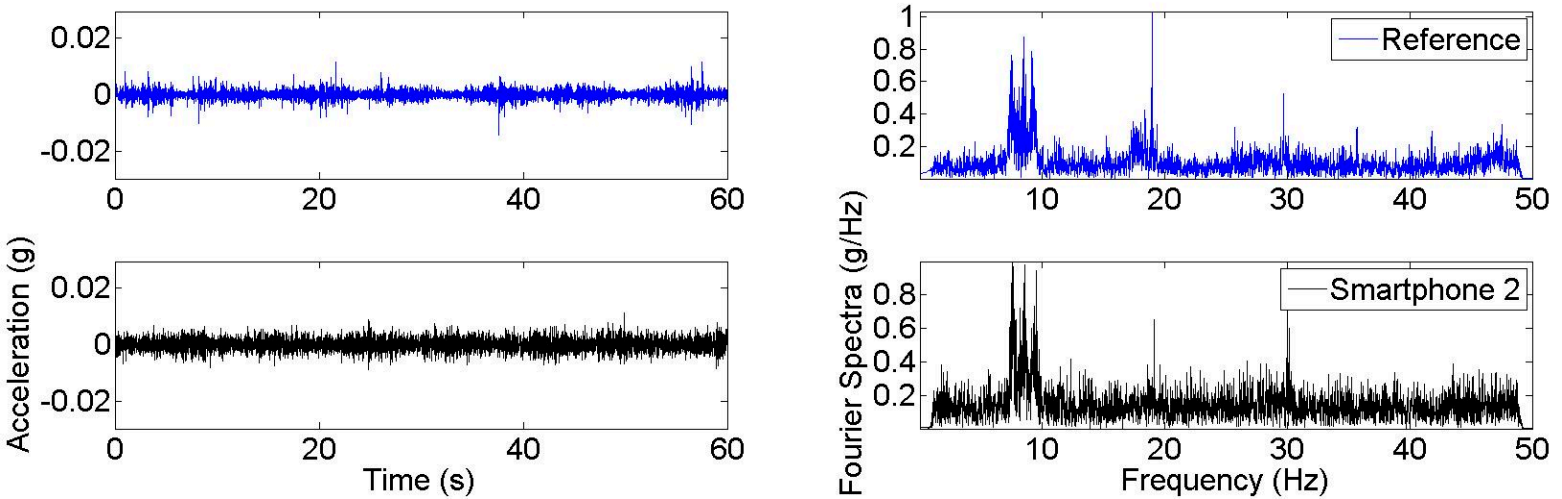
Acceleration time histories and Fourier spectra samples from pedestrian-induced vibrations.

In SHM practice, the dynamic load patterns might also have an effect on identification results. Test 1–6 are conducted under ambient vibration, which can be considered an output-only problem. On the contrary, operational loads such as pedestrian-induced or vehicle-induced vibrations might have dominant frequencies which will influence the identification results. For example, when a pedestrian walks on a bridge, the bridge is subjected to a harmonic vibration of approximately 1.6–2.4 Hz [59]. Therefore, pedestrian-induced vibrations tend to excite the modes which are close to 1.6–2.4 Hz. To reveal this effect, measurements are obtained under walking-induced vibration, and presented in Figure 12. Accordingly, it is seen that the 1st modal frequency, which is the closest frequency to pedestrian-induced frequencies, is significantly excited. This might explain why uncontrolled crowd dataset is dominated by Mode 1, whereas ambient vibration datasets frequently identify Mode 2 and Mode 3.

To summarize, the field measurement results show that the modal identification of the bridge structure can be conducted efficiently by the developed platform integrating citizens, new generation smartphones, and web-based server capabilities. In particular, as citizens continue providing more samples, the identification results will become more reliable and will provide useful information for big data generation. Using this platform, an online, remote, automated, secure and long-term monitoring system can be established and tested on multiple structures.

## 5. Conclusions

This study develops a novel SHM platform based on citizen crowdsourcing and smartphone sensors. The platform not only provides citizens with the necessary tools to measure and submit structural vibration using their smartphones, but also automatically processes the citizen-submitted data at a server to identify structural modal parameters (such as natural frequencies) useful for long-term structural health monitoring. A mobile application called *Citizen Sensors for SHM* was developed for measuring structural vibration with the smartphone-embedded accelerometers and submitting the data to the server. A web-based software package was developed for receiving and processing the citizen submission data on the server. Finally, the integrated system was evaluated on a real bridge structure using different smartphone generations under varying coupling and location conditions. High-fidelity accelerometers are also used as reference sensors. Low-amplitude ambient vibration of the bridge was measured by both the reference and smartphone sensors. The structural modal properties were identified and compared. The performance of the proposed platform and the test results can be summarized as follows:

(1) The developed platform is novel in the way it utilizes ubiquitous smartphones, crowdsourcing and citizen engagement as means of vibration-based SHM. It lays a foundation for a future citizen-centered cyber-physical sensor system for monitoring the integrity and safety of spatially-distributed urban infrastructure.
(2) Crowdsourcing-based SHM is a unique participatory sensing example in the way it synthesizes distinctive crowdsourcing parameters such as participatory and opportunistic involvement, multiple incentives, individual and collective data wisdom, and heterogeneous and homogeneous contribution with a hybrid human-sensor framework.
(3) Considering different generations of smartphone models, new generation smartphones provide better performance for vibration measurement. Time history data, Fourier spectra, and Arias intensity results show that as the phone generation gets younger, accuracy and sensitivity gets closer to the high quality reference measurements. In contrast, the oldest generation, Smartphone 1, is subjected to a high noise level which can mask structure’s dynamic characteristics in vibration signals. Although amplitude performance changes significantly according to the smartphone generation, modal identification results obtained from new generation smartphones have extremely small errors ranging around 1%, whereas the oldest generation, Smartphone 1, is incapable of identifying modal frequencies.
(4) The results show that the presented phone-structure coupling conditions did not affect monitoring performance significantly. On the other hand, such observation is likely to change as the vibration level gets higher than ambient vibration. Therefore, coupling effects under operational or extreme environmental vibrations can be different, and should be investigated in the future.
(5) Sensor location has an important effect on identification results, since modal displacements vary according to the measurement location. For instance, data submissions from one-sixth span identify the 3rd mode frequently, whereas the 2nd mode is dominant for other submission locations.
(6) Collecting a large number of small-sized vibration data submissions and averaging their frequency spectra can generate a useful database for crowdsourcing-based modal identification and monitoring purposes. This will enables the setup of a reliable large-sized database by small contributions from each citizen. In other words, retrieval of ubiquitous vibration data from smartphones enables identification of modal frequencies accurately without excessive citizen effort.
(7) The web platform provides secure but online, automated, remote, and widely accessible media for vibration data and modal identification results. What is more, the mobile platform provides users with the opportunity to choose the preferred communication tools, which means users can submit the data either instantaneously or when preferred communication tools are available.
(8) The proposed methodology is cost-effective and sustainable since the sensor instrumentation and maintenance is provided spontaneously by smartphone users. If the crowdsourcing model is improved, and the mobile application is distributed among the community, it can become an innovative source for long-term SHM applications.

Based on the conclusions drawn from this study, a long-term monitoring study will be initialized which only relies on smartphone data rather than high-quality reference sensing platforms. The smartphone application developed herein will be distributed throughout the community and strategies for citizen encouragement will be developed. Moreover, the applicability of the proposed system will be investigated for different structures. Improvement of the multilayered platform with further tests, and validation of the system with citizen participation will be a novel contribution to the smart cities concept. Eventually, as the database size increases exponentially in the long term and the application is extended to new structures, a big data model will be introduced to effectively handle the extensive data variety, velocity and volume.

## References

[1] Skolnik D., Lei Y., Yu E., Wallace J.W. (2006). Identification, model updating, and response prediction of an instrumented 15-story steel-frame building. Earthq. Spectra.

[2] Catbas F.N., Susoy M., Frangopol D.M. (2008). Structural health monitoring and reliability estimation: Long span truss bridge application with environmental monitoring data. Eng. Struct..

[3] Moaveni B., He X., Conte J.P., Restrepo J.I. (2010). Damage identification study of a seven-story full-scale building slice tested on the UCSD-NEES shake table. Struct. Saf..

[4] Gomez H.C., Fanning P.J., Feng M.Q., Lee S. (2011). Testing and long-term monitoring of a curved concrete box girder bridge. Eng. Struct..

[5] Ozer E., Soyoz S. (2015). Vibration-based damage detection and seismic performance assessment of bridges. Earthq. Spectra.

[6] Ozer E., Feng M.Q., Soyoz S. (2015). SHM-integrated bridge reliability estimation using multivariate stochastic processes. Earthq. Eng. Struct. Dyn..

[7] Carden E.P., Fanning P. (2004). Vibration based condition monitoring: A review. Struct. Health Monit..

[8] Wald D.J., Quitoriano V., Worden C.B., Hopper M., Dewey J.W. (2012). USGS “Did you feel it?” internet-based macroseismic intensity maps. Ann. Geophys..

[9] Cochran E., Lawrence J., Christensen C., Chung A. (2009). A novel strong-motion seismic network for community participation in earthquake monitoring. IEEE Instrum. Measur. Mag..

[10] Clayton R.W., Heaton T., Chandy M., Krause A., Kohler M., Bunn J., Aivazis M. (2012). Community seismic network. Ann. Geophys..

[11] Dashti S., Bray J.D., Reilly J., Glaser S., Bayen A., Mari E. (2014). Evaluating the Reliability of Phones as Seismic Monitoring Instruments. Earthq. Spectra.

[12] Morgenthal G., Höpfner H. (2012). The application of smartphones to measuring transient structural displacements. J. Civil Struct. Health Monit..

[13] Höpfner H., Morgenthal G., Schirmer M., Naujoks M., Halang C. (2013). On measuring mechanical oscillations using smartphone sensors: Possibilities and limitation. ACM SIGMOBILE Mob. Comput. Commun. Rev..

[14] Kohler M.D., Heaton T.H., Cheng M.H. (2013). The Community Seismic Network and Quake-Catcher Network: Enabling structural health monitoring through instrumentation by community participants. Proc. SPIE.

[15] Howe J. (2006). The rise of crowdsourcing. Wired Mag..

[16] Brabham D.C. (2008). Crowdsourcing as a model for problem solving an introduction and cases. Convergence.

[17] Albors J., Ramos J.C., Hervas J.L. (2008). New learning network paradigms: Communities of objectives, crowdsourcing, wikis and open source. Int. J. Inf. Manag..

[18] Hammon D.K.L., Hippner H. (2012). Crowdsourcing. Bus. Inf. Syst. Eng..

[19] Zhao Y., Zhu Q. (2014). Evaluation on crowdsourcing research: Current status and future direction. Inf. Syst. Front..

[20] Schenk E., Guittard C. (2011). Towards a characterization of crowdsourcing practices. J. Innov. Econ. Manag..

[21] Estellés-Arolas E., González-Ladrón-de-Guevara F. (2012). Towards an integrated crowdsourcing definition. J. Inf. Sci..

[22] Von Hippel E. (1976). The dominant role of users in the scientific instrument innovation process. Res. Policy.

[23] Corney J.R., Torres-Sánchez C., Jagadeesan A.P., Yan X.T., Regli W.C., Medellin H. (2010). Putting the crowd to work in a knowledge-based factory. Adv. Eng. Inform..

[24] Rossen B., Lok B. (2012). A crowdsourcing method to develop virtual human conversational agents. Int. J. Hum.-Comput. Stud..

[25] Kazman R., Chen H.M. (2009). The metropolis model a new logic for development of crowdsourced systems. Commun. ACM.

[26] Doan A., Ramakrishnan R., Halevy A.Y. (2011). Crowdsourcing systems on the world-wide web. Commun. ACM.

[27] Wallin M.W., Von Krogh G. (2010). Organizing for Open Innovation: Focus on the Integration of Knowledge. Organ. Dyn..

[28] Ebner W., Leimeister J.M., Krcmar H. (2009). Community engineering for innovations: The ideas competition as a method to nurture a virtual community for innovations. R&D Manag..

[29] Wu W., Tsai W.T., Li W. (2013). An evaluation framework for software crowdsourcing. Front. Comput. Sci..

[30] Boulos M.N.K., Resch B., Crowley D.N., Breslin J.G., Sohn G., Burtner R., Pike W.A., Jezierski E., Chuang K.Y.S. (2011). Crowdsourcing, citizen sensing and sensor web technologies for public and environmental health surveillance and crisis management: Trends, OGC standards and application examples. Int. J. Health Geogr..

[31] Fienen M.N., Lowry C.S. (2012). Social. Water—A crowdsourcing tool for environmental data acquisition. Comput. Geosci..

[32] Heipke C. (2010). Crowdsourcing geospatial data. ISPRS J. Photogramm. Remote Sens..

[33] Goodchild M.F., Glennon J.A. (2010). Crowdsourcing geographic information for disaster response: A research frontier. Int. J. Digit. Earth.

[34] Uden M., Zipf A. (2013). Open building models: Towards a platform for crowdsourcing virtual 3D cities. Progress and New Trends in 3D Geoinformation Sciences.

[35] Zhai Z., Hachen D., Kijewski-Correa T., Shen F., Madey G. Citizen engineering: Methods for “crowdsourcing” highly trustworthy results. Proceedings of the 2012 45th Hawaii International Conference on System Science (HICSS).

[36] Kijewski-Correa T., Su S., Montestruque L. A Citizen-Centric Health Monitoring Paradigm Using Embedded Self-Locating Wireless Sensor Networks. Proceedings of the 20th Analysis and Computation Specialty Conference.

[37] Hirth M., Hoßfeld T., Tran-Gia P. (2013). Analyzing costs and accuracy of validation mechanisms for crowdsourcing platforms. Math. Comput. Model..

[38] Goodchild M.F., Li L. (2012). Assuring the quality of volunteered geographic information. Spat. Stat..

[39] Reilly J., Dashti S., Ervasti M., Bray J.D., Glaser S.D., Bayen A.M. (2013). Mobile phones as seismologic sensors: Automating data extraction for the iShake system. IEEE Trans. Autom. Sci. Eng..

[40] Feng M., Fukuda Y., Mizuta M., Ozer E. (2015). Citizen Sensors for SHM: Use of Accelerometer Data from Smartphones. Sensors.

[41] Zheng H., Li D., Hou W. (2011). Task design, motivation, and participation in crowdsourcing contests. Int. J. Electron. Commer..

[42] Borst I. (2010). Understanding Crowdsourcing: Effects of Motivation and Rewards on Participation and Performance in Voluntary Online Activities.

[43] Zichermann G., Cunningham C. (2011). Gamification by Design: Implementing Game Mechanics in Web and Mobile Apps.

[44] Kapp K.M. (2012). The Gamification of Learning and Instruction: Game-Based Methods and Strategies for Training and Education.

[45] Ozer E. Analysis Report for Structural System Identification and Acceleration Record Manager. http://ekinstitute.com/analiz.html.

[46] Ozer E. Design Report for Structural System Identification and Acceleration Record Manager. http://ekinstitute.com/tasarim.html.

[47] Ozer E. Implementation Report for Structural System Identification and Acceleration Record Manager. http://ekinstitute.com/gerceklestirim.html.

[48] Ray J. (2012). Sams Teach Yourself iOS 5 Application Development in 24 Hours.

[49] Allan A. (2011). Basic Sensors in iOS: Programming the Accelerometer, Gyroscope, and More.

[50] Ozer E. Citizen Sensors for SHM: iPhone Application. https://itunes.apple.com/us/app/citizen-sensors-for-shm/id986036957?mt=8.

[51] Neuburg M. (2013). Programming IOS 7.

[52] Neuburg M. (2013). iOS 7 Programming Fundamentals: Objective-c, Xcode, and Cocoa Basics.

[53] Ozer E. Structural System Identification and Acceleration Record Manager. http://www.ekinstitute.com.

[54] Oppenheim A.V., Schafer R.W. (2009). Discrete-Time Signal Processing.

[55] Welling L., Thomson L. (2003). PHP and MySQL Web Development.

[56] Chatzimilioudis G., Konstantinidis A., Laoudias C., Zeinalipour-Yazti D. (2012). Crowdsourcing with smartphones. IEEE Internet Comput..

[57] Brincker R., Zhang L., Andersen P. (2001). Modal identification of output-only systems using frequency domain decomposition. Smart Mater. Struct..

[58] Arias A., Hansen R.J. (1970). A Measure of Earthquake Intensity, Seismic Design for Nuclear Power Plants.

[59] Bachmann H. (1992). Case studies of structures with man-induced vibrations. J. Struct. Eng..

